# Identifying with a Classically Liberal Nation: A Social Justice Perspective on Majority Opposition to Multiculturalism

**DOI:** 10.5334/irsp.941

**Published:** 2025-03-19

**Authors:** Jessica Gale, Antoine Roblain, Christian Staerklé

**Affiliations:** 1 University of Lausanne, CH; 2 UniversitéLibre de Bruxelles, BE

**Keywords:** Multiculturalism, Social justice, National identification, Majorities, Individual responsibility

## Abstract

Opposition to multiculturalism is common among native majorities. Normatively, this group-based political theory and public policy has been described as being incompatible with the individual justice-based orientation of Western liberal societies. In this research, we account for national majority opposition to multiculturalism by arguing that national identities in classically liberal societies are primarily associated with individual justice beliefs, in opposition to group-based justice beliefs. A correlational (*N* = 91) and an experimental (*N* = 172) study in Switzerland first show that the relationship between national identification and opposition to multiculturalism is partially explained by a belief in individual responsibility, a key facet of individual justice. This result was replicated using representative Swiss data from the World Values Survey (*N* = 1241), as well as in Belgium (*N* = 362), another Western liberal society. Effects transcended an ethnic conception of national identity and provide a novel perspective on majority multicultural attitudes as rooted in group-based conceptions of social justice.

Throughout Europe, opposition to multiculturalism has increased over recent decades. This opposition has been coupled with the rise of populist right-wing political movements (Müller, 2016), prioritizing ‘natives’ over newcomers and favoring cultural homogeneity (Staerklé & Green, 2018). Indeed, opposition to multiculturalism is often reported among those who see themselves as members of native majorities (Verkuyten, 2009).

Existing research suggests that native majorities feel excluded from the multicultural emphasis on cultural minority rights, needs and distinctiveness (Plaut et al., 2011). They may also feel that their dominant position and identity within the national society is threatened by policies that compensate minority disadvantages and that recognize and promote minorities’ culture-specific festivals, holidays and languages (Badea et al., 2018; Rios, 2022; Yogeeswaran & Dasgupta, 2014b). From the perspective of normative political theory, an additional argument against multiculturalism suggests that this group-based political theory and public policy is incompatible with the individualistic orientation of Western liberal societies (Joppke, 2004; Kymlicka, 2013). In this article, we argue that ideological belief systems associated with national identity that give priority to individual forms of justice may explain opposition to multiculturalism by national majorities. In doing so, we provide a novel perspective on multicultural attitudes that is rooted in fundamental justice belief systems (Staerklé, 2009; Tyler & van der Toorn, 2013) that are shaped by the groups with which people identify.

In the following, we first define multiculturalism in line with group-based forms of justice. We then outline the research, focusing on how the *content* of national identity has implications for intergroup attitudes and support or opposition to group-based forms of justice. Finally, we introduce the role played by dominant individual justice beliefs associated with Western national identity.

## Abstract versus Concrete Multiculturalism

Multiculturalism can be defined both as an abstract prescriptive ideology that explicitly recognizes and values group-based cultural differences, and as a concrete public policy that seeks to compensate disadvantages experienced by cultural minority groups (Moghaddam, 2008; Sears et al., 1999; Yogeeswaran & Dasgupta, 2014b). Both definitions are based on collective justice principles, in which categorical differentiation between national (sub-)groups drives justice perceptions and where treatment of (sub-)groups in accordance with their particular needs and disadvantages is imperative (Azzi, 1992; Gale & Staerklé, 2019; Gale & Yogeeswaran, 2024; Green & Staerklé, 2023). Research has demonstrated that for national majorities, abstract construals of multiculturalism are less controversial than concrete multicultural policies, with tangible policies leading to greater perceived threat, and thus to greater opposition (Yogeeswaran & Dasgupta, 2014b; see also Sears et al., 1999). In our studies, we consider both abstract (ideology) and concrete (policy) facets of multiculturalism, expecting that national majority members who identify with the nation should be more opposed to its policy than to its ideology.

## Content of National Identity

The meaning associated with national identity—revealed through its content—should shape the degree to which it predicts opposition, or support, for multiculturalism (Yogeeswaran & Dasgupta, 2014a, for an overview). In the Netherlands, for example, national identification has been defined by feelings of national nostalgia and autochthony (that is, a belief that a place belongs to its original/historic inhabitants who are therefore more entitled than newcomers). There, national identification has been shown to be associated with stronger prejudice and opposition to multiculturalism (Martinovic & Verkuyten, 2013; Smeekes et al., 2015). In a similar way, political debates regarding newcomers in assimilationist countries such as Switzerland ‘can be interpreted as a politics of national identity’ (Riaño & Wastl-Walter, 2006, p. 17; see also Politi et al., 2020), as exemplified by political discourse demonstrating an exclusionary stance towards newcomers in the name of ‘protecting’ the nation’s identity. Conversely, when a nation’s identity is defined by diversity itself—that is, when multiculturalism is part of the group’s norms and cultural minorities are included in the national concept, such as in Canada—stronger national identification is associated with decreased prejudice and increased support for multiculturalism (Devos & Mohamed, 2014; Esses et al., 2006; Reynolds et al., 2015).

Similarly, the classic distinction between civic versus ethnic conceptions of citizenship and nationhood have been shown to differentially affect attitudes towards newcomers (Brubaker, 1990; Pehrson & Green, 2010). Indeed, newcomers are perceived and received more positively in countries that place greater importance on residents’ legal citizenship and active participation in society than on blood relations, race, ethnicity, language or cultural tradition (see also Reijerse et al., 2015). In other words, a link between national identity and negative outgroup attitudes is more prevalent in countries with an ethnic, homogenizing, and essentialist definition of national identity, as opposed to countries with a more civic definition that is, at least in principle, more inclusive (see also Ariely, 2012).

Yet, civic conceptions can still be assimilationist and exclusionary if they are not supplemented with the normative value of cultural diversity (Soutphommasane, 2005). For example, a civic conception of citizenship implies that all members of society are expected to contribute to and be committed to the national community, whereas the celebration of cultural diversity and ethnic origins may be left to the private sphere rather than actively (politically and/or normatively) endorsed. Indeed, a key component of the civic conception is its pervasive focus on individual responsibility, regardless of (cultural) group membership. If national majorities show opposition to multiculturalism in such societies, it is likely (at least in part) because they adhere to these shared beliefs (i.e., norms) of individual justice that are central to their national identity and that they perceive to be incompatible with multiculturalism.

## National Identity and the (Nationally) Shared Belief in Individual Responsibility

According to a basic principle of Social Identity Theory (Tajfel & Turner, 1979), identifying with a group implies endorsement of its norms that thereby act as a guide for attitudes and behavior (see also Reicher et al., 2010). Somewhat paradoxically, however, Jetten et al. (2002) showed that in individualist cultures of Western countries, stronger national identification implies greater adherence to the norm that ‘we are all individuals’. As a result, dominant group members perceive themselves as ‘default’ individuals rather than as group members (Deschamps, 1982; Iacoviello & Lorenzi-Cioldi, 2015). This individualistic norm is consistent with the tendency in Western liberal countries to attribute individuals’ behavior and choices to their internal characteristics rather than to situational factors (i.e., fundamental attribution error; Ross, 1977), underlying individual justice principles. Through such an individualizing lens, (cultural) minority members are easily perceived as deviating from a common, national norm rather than as members of groups with alternative norms and ways of life (Staerklé, 2009). As van Oorschot (2006) suggests, immigrants are at the bottom of the (welfare) ‘deservingness ranking’ in every European country (see also Laenen & Meuleman, 2017), as they are often ‘blamed’ for their ‘neediness’, and considered personally responsible for challenging life situations. The endorsement of this individualistic norm, which gives rise to individual justice principles of personal responsibility (and relatedly merit, hard work, equity between individuals, also aligned with ‘colorblind’ ideology; Gale & Yogeeswaran, 2025), can therefore be a paradoxical way of showing loyalty to one’s (national) group, and an argument to reject diversity.

At the level of political theory, the classically liberal emphasis on individual responsibility is in tension both with interventionist, redistributive measures (related to multicultural policy; Kukathas, 2003), and with a communitarian, group-based vision of society (see also Shweder et al., 1997).^1^ Empirical research among majorities is consistent with this liberal political theory and discourse and confirms, for example, that group-based policies such as affirmative action are perceived as violating principles of equal opportunity and individual responsibility (Bobocel et al., 1998; Son Hing et al., 2011). Gale and Staerklé (2019) furthermore showed that when dominant norms of individual responsibility are salient in classically liberal societies, dominant (e.g., national) majorities tend to express opposition to multiculturalism and justice between groups.

The dominant norm of individual responsibility should therefore become salient when people identify with a classically liberal nation. Accordingly, the present research tested whether national identification of majority members is associated with increased adherence to individual justice norms, and whether this increase is in turn associated with opposition to both multicultural policy and perhaps ideology, too.

## The Present Studies

The present studies were conducted primarily in Switzerland, an economically liberal country where individual justice beliefs (expressed in endorsement of individual freedom, responsibility, and deservingness) are prevalent (Schwiter, 2013). According to the index of economic freedom (The Heritage Foundation, 2023), Switzerland is the most economically liberal country in Europe, defined by strong property rights, flexible labor regulations, low taxes, and relatively low government spending. Hence, while many other countries share its liberal emphasis on individuals, Switzerland is a *prototypically* (economically and *classically*) liberal nation (Wimmer, 2011). We therefore expected individual justice beliefs to vary as a function of the level of national identification: the more individuals identify with Switzerland, the more they should endorse individual justice beliefs.

We also acknowledge that variation may exist in other Western contexts where a normative emphasis on individuals may be coupled with greater normative endorsement of diversity. This coupling is arguably the case in some Western liberal settler societies like Canada, or in some European countries with less exclusionary histories or that have undergone significant constitutional amendments to enable an inclusionary stance towards diversity. In such contexts, adherence to individual justice beliefs should still vary depending on national identification, but these beliefs may be less associated with opposition to multiculturalism.

Four studies were conducted to examine the role played by national identification and beliefs in individual justice in attitudes towards multiculturalism. Our general hypothesis was that national identification should be associated with opposition to multiculturalism via individual justice beliefs; an indirect pathway. In other words, stronger national identification should be associated with increased individual justice beliefs (H1a), which, in turn, should be associated with opposition to multiculturalism (H1b). Study 1 featured a correlational design, measuring national identification and testing if a stronger feeling of attachment to the country is associated with a stronger belief in individual responsibility, thereby explaining increased opposition to multiculturalism. Study 2 adopted a fictitious society experimental design to test causality of the link between individual justice beliefs and opposition to multiculturalism. Study 3 sought to demonstrate the hypothesized process with nationally representative survey data. Study 4 tested the same hypothesized process in an alternate Western and less prototypically liberal country: Belgium. All studies adhere to the APA Code of Conduct ethical guidelines, including obtaining informed consent from all participants.

## Study 1

### Method

#### Participants

Participants were recruited via an online snowball technique in French-speaking Switzerland (*N* = 141).^2^ To ensure a sample of national majority group members, those with Swiss citizenship only were retained (*N* = 91).^3^ The mean age was 26.93 years (*SD* = 10.47; 85% under 30 years old) and 68% (*N* = 62) were female.

#### Procedure and Materials

Participants were invited via social media to participate in a study on ‘life in society’. The online questionnaire was composed of the following measures, all assessed on a 1 (*strongly disagree*) to 6 (*strongly agree*) scale, as well as socio-demographic questions.

**National Identification** was measured with four items (α = .88; Falomir-Pichastor & Frederic, 2013), e.g., ‘I identify with Switzerland’ (all items in Supplementary File 1).

**Individual Responsibility Belief** was measured with four items (α = .78; Major et al., 2002) attributing individuals’ progress to their own responsibility, e.g., ‘Most people who do not progress should not blame the system; they are responsible themselves.’

**Support for Multicultural Ideology** was measured with four items (α = .77; Berry & Kalin, 1995; Guimond et al., 2014; Ryan et al., 2007), e.g., ‘Cultural affiliation is a precious distinction between individuals that should be valued.’

**Support for Multicultural Policy** was measured with three items reflecting affirmative action measures in favor of cultural minorities (α = .79; Gale & Staerklé, 2019), e.g., ‘In order to guarantee a certain diversity between employees, positions should be reserved for qualified members of cultural minority groups.’

### Results

Data analysis was carried out using SPSS, version 29. Descriptive statistics were assessed first, followed by hypothesis testing. To retain the complete sample of respondents and to prevent a reduction in statistical power, Maximum Likelihood estimation was applied for missing cases of items representing the four measures (3.8% missing data).

#### Descriptive Statistics and Correlations

Table 1 shows that participants strongly identified with Switzerland, and the belief in individual responsibility was close to the scale mid-point. While participants were fairly supportive of multicultural ideology, they were significantly less so of its policy, *t*(90) = 9.83, *p* < .001, *d* = 2.07, even though the two were strongly correlated. Those who strongly identified with Switzerland showed significantly less support for multicultural policy, but not ideology. In line with H1a, stronger feelings of national identification were associated with a stronger belief in individual responsibility, and in line with H1b, a stronger belief in individual responsibility was associated with weakened support for both multicultural ideology and policy.

**Table 1:** Means, Standard Deviations and Bivariate Correlations between Variables (Study 1).


	*M*	*SD*	2.	3.	4.

1. National Identification	4.77	1.01	.42***	–.41***	–.06

2. Individual Responsibility	3.21	0.98		–.61***	–.36**

3. Multicultural Policy	3.11	1.17			.49***

4. Multicultural Ideology	4.24	0.96			


*Note*. ***p < .001, **p < .01.

#### Hypothesis Testing via Indirect Effects

Two models testing indirect effects were calculated using PROCESS v4.2 (Hayes, 2022) with support for multicultural policy and ideology as outcome variables. National identification was the predictor variable and belief in individual responibility was the intermediary variable in both models. Covariates included age and gender.^4^

**Figure 1:**
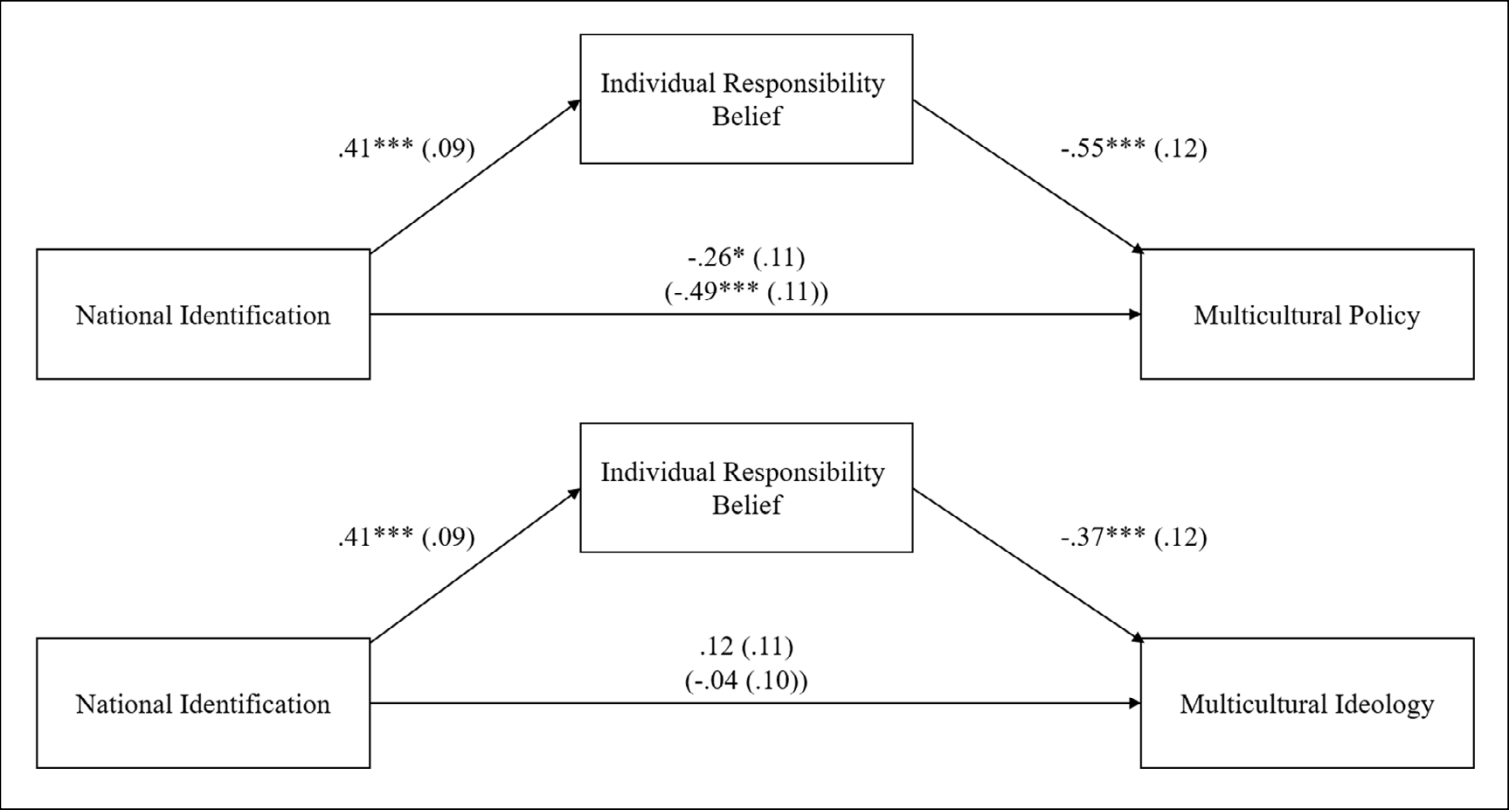
Negative Indirect Effects of National Identification on Support for Multicultural Policy, and Ideology, via Individual Responsibility Belief (Study 1).

Figure 1 shows unstandardized regression coefficients and standard errors for both models. Consistent with H2a, stronger national identification was associated with increased individual responsibility belief, 95% CI [0.24, 0.59], *t*(87) = 4.64, *p* < .001, *d* = 0.99. Highly identified participants had a stronger belief that individuals were responsible for their own fate, demonstrating adherence to individual justice principles.

Consistent with H1b, a stronger belief in individual responsibility was related to significantly lower support for both multicultural policy and ideology, 95% CI [–0.78, –0.32], *t*(86) = –4.79, *p* < .001; *d* = 1.03; 95% CI [–0.60, –0.14], *t*(86) = –3.21, *p* = .002, *d* = 0.69, respectively. The more participants believed individuals were responsible for their own fate, the less they supported multiculturalism.

The indirect effect of national identification on support for multiculturalism through individual responsibility belief was significant in both models, *B* = –.23, *SE* = .07, *95% CI* [–0.39, –0.11] multicultural policy, *B* = –.15, *SE* = .07, *95% CI* [–0.30, –0.04] multicultural ideology. The more citizens identified with Switzerland, the greater their endorsement of the dominant norm of individual responsibility, and the more they opposed multiculturalism.^5^

### Discussion

The results from Study 1 provide preliminary evidence that a belief in individual responsibility explains why nationals who have a strong sense of national identification may reject multiculturalism. Indeed, this belief expresses individual justice principles that are dominant in many Western nations and are thus supported by national identification (Jetten et al., 2002). From the perspective of national majorities in a classically liberal society, this belief stands in opposition to multiculturalism (i.e., collective justice principles).

The purpose of our model was to show the conceptual *linkages* between national identification, the belief in individual responsibility, and multicultural attitudes. However, indirect effects imply causality, and one could question the directionality of our conceptual model: Is it national identification that shapes a belief in individual responsibility, which ultimately leads to a rejection of multiculturalism? Or could a shared belief in individual responsibility instead lead people to identify more strongly with their country? And does the belief in individual responsibility indeed precede (rather than succeed) a rejection of multiculturalism?

## Study 2

To provide experimental evidence for the hypothesized process associating individual justice beliefs and opposition to multiculturalism, Study 2 adopted a fictitious island paradigm (Azzi, 1992; Gale & Staerklé, 2019; Gale & Yogeeswaran, 2025) in which participants imagined themselves as members of a national majority group living on an island characterized by either strong or weak shared beliefs in individual responsibility. This methodological approach allowed an examination of a social context similar to—yet simplified from—the one participants experience in their real lives in Switzerland. It also allowed us to control the aspects of society we wanted participants to pay attention to, specifically concerning shared beliefs in individual responsibility that are (in the real world) rather dominant in Switzerland.

We expected multiculturalism to be significantly less supported in a fictitious society characterized by strong shared beliefs in individual responsibility, compared with a fictitious society characterized by weak shared beliefs in individual responsibility (H1b). We also checked whether identification with the island would be associated with individual responsibility beliefs.

Because of the nature of the experiment, in this study we opted to include all participants regardless of their citizenship. However, we included citizenship as a potential moderator, assuming the predicted effects may still be most pronounced among participants with Swiss citizenship only. This would be the case because native majorities form the group for whom our overarching hypothesis applies (assuming those with other citizenships maintain some ties outside the country, thus affecting their shared beliefs and views on multiculturalism). Moreover, because real-world conditions may easily transpose onto perceptions of fictitious societies (Azzi, 1992; Gale & Staerklé, 2019; Gale & Yogeeswaran, 2025), this procedure allowed us to verify if our hypothesis applies, indeed, only for (real) national (native) majorities.

### Method

#### Participants

Participants were students from an introductory social psychology class in French-speaking Switzerland (N = 172). The mean age was 20.71 (*SD* = 4.02) and 83.1% (*n* = 143) were female. Those with Swiss citizenship only comprised 43.0% of the sample (*n* = 74), while the remainder were either non-Swiss (19.8%, *n* = 34), or were naturalized and/or had other citizenships, too (36.6%; *n* = 63).^6^

#### Procedure and Materials

Participants were invited to fill out a questionnaire on social issues that involved some imagination. They were first asked to read a text that described an island inhabited by several groups and then to imagine themselves as a member of the majority group (native to the island; having lived there forever). Most of the text was kept constant (suggesting, for example, that some minority groups were less well-off than the majority), and participants were randomly assigned to one of two conditions. In the first condition, participants read that most inhabitants thought that everyone could get ahead in society depending on their own will and motivation, and that the value of individual responsibility was therefore very important to inhabitants (high individual responsibility condition, *n* = 87). In the second condition, participants read that most inhabitants thought that some people could not get ahead in society as a function of their own will and motivation, and that therefore the value of individual responsibility had little importance to inhabitants (low individual responsibility condition, *n* = 85).

At the end of the description, they read that experts mandated by the government had recently proposed measures to improve the social situation on the island. Participants were then asked to respond to a series of questions while still imagining themselves as a member of the majority group: the same multicultural policy items from Study 1, including a fourth item (α = .80), and the same multicultural ideology items from Study 1, including a fifth item (α = .64; see Supplementary File 1).

Subsequently, the manipulation check included two items that assessed individual responsibility belief (the first and third items from Study 1; *r* = .51).^7^ Participants were also asked to indicate the degree to which they identified with the island society described in the text (single item; Postmes et al., 2013).

### Results

#### Descriptive Statistics and Correlations

Table 2 shows descriptive statistics for each condition separately as well as general bivariate correlations between all variables in the study. The only significant difference between the two experimental conditions was the belief in individual responsibility, *t*(168) = 2.71, *p* = .003, *d* = 0.42. Differences between means for all other measures were non-significant (national identification *t*(169) = –1.17, *p* = .242, *d* = 0.18; support for multicultural policy *t*(169) = 0.90, *p* = .367, *d* = 0.14; support for multicultural ideology *t*(168) = 1.02, *p* = .310, *d* = 0.16).

**Table 2:** Means, Standard Deviations (by Experimental Condition) and Bivariate Correlations between Variables (Study 2).


	HIGH INDRESP CONDITION	LOW INDRESP CONDITION			

	*M (SD)*	*M (SD)*	2.	3.	4.

1. National Identification	4.64 (1.44)	4.38 (1.49)	.25*	–.14	.15*

2. Individual Responsibility	4.01 (1.36)	3.48 (1.18)		–.21**	.08

3. Multicultural Policy	4.03 (1.21)	4.20 (1.08)			.33***

4. Multicultural Ideology	4.26 (0.94)	4.39 (0.71)			


*Note*. ***p < .001, **p < .01, *p < .05, ^*p* < .10.

Correlations between variables were partially consistent with those in Study 1. Stronger identification with the island was associated with a stronger belief in individual responsibility, weaker support for multicultural policy, and stronger support for multicultural ideology. Moreover, a stronger belief in individual responsibility was associated with weaker support for multicultural policy, but not ideology.

#### Manipulation Checks

Consistent with descriptive statistics above, when controlling for age and gender, a one-way analysis of covariance (ANCOVA) confirmed that participants in the high individual responsibility condition (*M* = 3.99, *SE* = 0.14) scored significantly higher on individual responsibility belief than those in the low individual responsibility condition (*M* = 3.48, *SE* = 0.14), *F*(1, 165) = 6.87, *p* = .010, η_p_^2^ = 0.04.

#### Hypothesis Testing

To test our hypotheses when including citizenship as a moderator, we calculated two models using PROCESS v4.2 (Hayes, 2022). Support for multicultural policy and ideology were outcome variables, and experimental conditions, citizenship, and the interaction between the two were predictors. Covariates included age and gender.

Neither citizenship nor experimental conditions revealed significant main effects on support for either multicultural policy or ideology. However, a significant interaction was found between citizenship and experimental conditions when predicting support for multicultural policy, *B* = –.40, *SE* = .17, *95% CI* [–0.74, –0.06], *t*(163) = –2.32, *p* = .021, Δ*R^2^* = 0.03. Decomposition suggests that among participants with Swiss citizenship only, and in line with H1b, those in the high individual responsibility condition expressed significantly less support for multicultural policy than those in the low individual responsibility condition, *B* = –.27, *SE* = .13, *95% CI* [–0.53, –0.01], *t*(163) = –2.08, *p* = .039, *d* = 0.33. For participants with other nationalities, the experimental conditions had no effect, *B* = .13, *SE* = .12, *95% CI* [–0.10, 0.36], *t*(163) = 1.15, *p* = .021, *d* = 0.18. No interaction effect was found for multicultural ideology, *B* = .09, *SE* = .13, *95% CI* [–0.16, 0.35], *t*(163) = 0.73, *p* = .468, Δ*R^2^* = 0.00.

### Discussion

The results of Study 2 suggest that the presence of strong shared beliefs in individual responsibility leads national majorities to reject multiculturalism, at least concerning multicultural policy (not ideology). This result may reflect the status-legitimizing nature of individual responsibility beliefs. Our theorizing about this belief suggests it can indeed serve to justify the social hierarchy (Joffe & Staerkle, 2007) and to frame *cultural minorities* as being undeserving of redistribution, thus explaining opposition to multiculturalism. Nevertheless, the belief in individual responsibility should not be reduced to its status-legitimizing function. Indeed, this belief reflects individual justice principles inherent to classical liberalism that imply a sense of agency in the face of injustice (Shweder et al, 1997) – principles widely considered incompatible with multiculturalism from a national majority perspective.

The present study also supports the idea that this belief in individual responsibility forms the basis of classically liberal cultural norms, (causally) shaping views on multiculturalism. We did not find an effect on multicultural ideology in this study, which is rather unsurprising. In the experimental manipulation, we made salient the culturally diverse nature of the island, involving respectful intergroup relations and the presence of different group-based traditions, languages, and religions. This normative aspect would have undoubtedly led participants to endorse recognition of cultural minorities independently of individual responsibility beliefs, as would be the case in societies that normatively endorse cultural diversity in addition to individual justice and classical liberalism (Devos & Mohamed, 2014; Esses et al., 2006; Reynolds et al., 2015; Soutphommasane, 2005). Furthermore, identification with the island and support for multicultural ideology were positively correlated, providing convergent evidence for this interpretation. Nevertheless, such a norm did not undermine that the shared belief in individual responsibility was associated with opposition to a more concrete and contentious multicultural policy.

The fictitious island paradigm adopted in this study allowed us to experimentally manipulate the perception of shared beliefs in individual responsibility, allowing for greater confidence in the directionality of our conceptual model.

## Study 3

A limitation of Studies 1 and 2 was the relatively small convenience samples. This led us to extend and replicate the findings with nationally representative survey data. Because secondary survey research involves the use of pre-existing measures, the items used in this third study were different from those in Studies 1 and 2, but they tapped similar and equivalent constructs.

### Method

#### Participants

World values survey data from Switzerland (wave 5, 2007) was used. The sample included 1241 participants, of which the majority were female (55.1%; *N* = 648), and the mean age was 52.45 (*SD* = 16.14). No participants were excluded from the study as there was no direct question in the Swiss data asking participants to indicate their citizenship (WVS Methodology Questionnaire Switzerland, 2007).^8^

#### Procedure and Materials

**National Identification** was measured with a single item, assessed on a scale from 1 (*strongly disagree*) to 4 (*strongly agree*): ‘I see myself as a citizen of Switzerland.’

**Hard Work Belief** was measured with a single item that asked the degree to which participants believed that ‘hard work doesn’t generally bring success, it’s more a matter of luck’ (*coded 1*), or that ‘in the long run, hard work usually brings a better life’ (*coded 10*). Similar to the Individual Responsibility Belief measure from Studies 1 and 2, this measure attributed an individual’s progress to their own responsibility (i.e., hard work) rather than luck. A high score captures a belief in individual responsibility and sensitivity to individual justice norms.

**Support for Multiculturalism** was measured with a single item in which participants indicated the degree to which they believed ‘ethnic diversity erodes a country’s unity’ (*coded 1*), or that ‘ethnic diversity enriches life’ (*coded 10*). This is a more abstract measure of support for multiculturalism than those used in the first two studies but still reflects sensitivity to collective justice principles. A high score signals a generally positive view of cultural diversity (more concrete measures were not available in this data).

### Results

Maximum Likelihood estimation method was applied for missing cases of items representing national identification, hard work, and support for multiculturalism (1.1% missing data).

#### Descriptive Statistics and Correlations

Participants had a strong sense of identification with Switzerland (*M* = 3.25, *SD* = 0.70), and a moderate belief that hard work brings a better life (*M* = 5.47, *SD* = 2.68), consistent with Studies 1 and 2. They were fairly supportive of multiculturalism (*M* = 6.78, *SD* = 2.09), demonstrating general adherence to the belief that ethnic diversity is enriching rather than eroding social life. Those who strongly identified with Switzerland expressed less support for multiculturalism (*r* = –.11, *p* < .001). Supporting H1a, stronger national identification was also associated with a stronger belief in the benefits of hard work (*r* = .15, *p* < .001), and supporting H1b, a stronger belief in hard work was associated with less support for multiculturalism (*r* = –.12, *p* < .001). These correlations generally align with Studies 1 and 2 (with some exceptions in Study 2, as discussed above).

#### Hypothesis Testing via Indirect Effect

Our hypothesized indirect effect was tested using PROCESS v4.2 (Hayes, 2022). Support for multiculturalism was the outcome variable, national identification was the predictor variable and hard work belief was the intermediary variable. Covariates included age and gender.^9^

**Figure 2:**
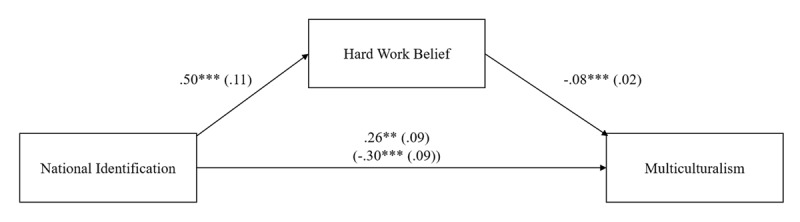
Negative Indirect Effect of National Identification on Support for Multiculturalism via Belief in Hard Work Belief (Study 3).

Figure 2 shows unstandardized regression coefficients and standard errors for the model. Consistent with H1a, stronger national identification was associated with increased hard work belief, 95% CI [0.28, 0.71], *t*(1237) = 4.52, *p* < .001, *d* = 0.26. Consistent with H1b, a stronger belief in hard work was associated with less support for multiculturalism, 95% CI [–0.13, –0.04], *t*(1236) = –3.67, *p* < .001; *d* = 0.21. The indirect effect of national identification on support for multiculturalism through hard work belief was significant as well, *B* = –.04, *SE* = .02, *95% CI* [–0.07, –0.02]. Consistent with Studies 1 and 2, the more individuals identified with Switzerland, the more they endorsed individual responsibility beliefs, and the more they opposed multiculturalism.

## Study 4

The focus on Switzerland in the previous studies raises the question as to whether this country is unique in supporting our hypothesized model. We therefore tested our model in Belgium, another Western European, classically liberal society with a variety of characteristics similar to those of Switzerland, but with a colonial past that may account for greater normative endorsement of cultural diversity and multiculturalism than Switzerland (see Banting & Kymlicka, 2013; like the fictitious society in Study 2). This study therefore permitted both a replication of the main hypothesized model in Belgium, and an examination of the findings from the fictitious island paradigm (Study 2) in real life. In this study, we manipulated individual responsibility beliefs associated with national identity to increase our confidence in the predicted causal effect. We also examined whether our hypothesized process associating national identification, individual justice beliefs and opposition to multiculturalism stands above and beyond the potential effect of an ethnic conception of national identity.

### Method

#### Participants

Participants were recruited from a Psychology student pool at a French-speaking university in Belgium (*N* = 391). The initial intention was to examine a sample of only national majority group members (https://aspredicted.org/W9N_C6S). However, as in Study 2, we decided to maintain the full sample, excluding only participants who failed attention checks (final *N* = 362),^10^ to investigate if our hypothesized effects apply specifically to national majority group members. The mean age was 20.46 (*SD* = 4.26) and 84.0% of participants (*n* = 304) were female. Belgian citizen only comprised 52.6% of the sample (*n* = 191), while the remainder were either non-Belgian (20.1%, *n* = 73), or had other citizenships, too (27.0%; *n* = 98).

#### Procedure and Materials

An experimental questionnaire was administered online and participants were randomly assigned to one of three conditions. In two conditions, participants were asked to read a fictitious newspaper article that reported details of a recent study on Belgian identity, that is, how most Belgians describe their traits and values. In one condition, participants read that most Belgians described themselves as autonomous individuals who are responsible for their own fate and progress (high individual responsibility condition; *n* = 112). In the second condition, participants read that very few Belgians (less than 5%) describe themselves this way (low individual responsibility condition; *n* = 125). In the third condition, no text was read (empty control condition; *n* = 125).

Following the experimental manipulation, participants responded to a series of questions coded on the same scale from Studies 1 and 2, ranging from 1 *(strongly disagree)* to 6 *(strongly agree)*. First, they were asked to indicate the degree to which they identified with Belgium (single item; Postmes et al., 2013), followed by the Belief in Individual Responsibility (four items from Study 1; α = .67), Support for Multicultural Policy (four items from Study 2; α = .82), and Support for Multicultural Ideology (five items from Study 2; α = .57).^11^ Subsequently, a measure of Ethnic Conceptions of National Identity was included (three items, α = .66; Meeus et al., 2010), followed by an open-ended question that asked participants to describe the values and traits they think define Belgians. Finally, prior to sociodemographic questions, participants who had read a fictitious newspaper article were asked to indicate whether the text suggested ‘the majority’ or, instead, ‘very few’ Belgians described themselves as individuals responsible for their own fate. Those who did not respond appropriately to this question (*n* = 29) were excluded, as indicated above.

### Results

#### Descriptive Statistics and Correlations

Table 3 shows a moderate level of national identification, and a belief in individual responsibility below the scale mid-point. Participants were somewhat supportive of both multicultural ideology and policy, with the former significantly more supportive than the latter, *t*(361) = 3.22, *p* < .001, *d* = 0.34. The two were strongly correlated with each other. In the overall sample, Belgian national identification was neither associated with endorsement of multicultural policy, nor ideology. However, supporting H1a, stronger national identification was associated with a stronger individual responsibility belief, and supporting H1b, a stronger individual responsibility belief was associated with weakened support for multicultural policy, but not ideology. Finally, an ethnic conception of Belgian national identity was positively associated with individual responsibility belief, justifying its inclusion in our conceptual model.

**Table 3:** Means, Standard Deviations, and Bivariate Correlations between Variables for the Full Sample in Belgium (Study 4).


	*M (SD)*	2.	3.	4.	5.

1. National Identification	3.80 (1.36)	.10*	–.02	–.02	.04

2. Individual Responsibility	2.71 (0.85)		–.27***	–.05	.23***

3. Multicultural Policy	4.03 (1.12)			.37***	–.10

4. Multicultural Ideology	4.22 (0.81)				–.02

5. Ethnic Conception	2.51 (1.11)				


*Note*. ***p < .001, **p < .01, *p < .05.

#### Preliminary Analyses

Controlling for age and gender, a one-way ANCOVA revealed differences in national identification between conditions, *F*(2, 354) = 4.14, *p* = .017, η_p_^2^ = 0.02. A Bonferroni-adjusted posthoc test showed that participants in the low individual responsibility condition (*M* = 3.54, *SD* = 1.38) expressed lower levels of national identification than those in the empty control condition (*M* = 4.03, *SD* = 1.37), Δ*M* = –0.49, *SE* = 0.17, *p* = .013. Participants in the high individual responsibility condition were in between (*M* = 3.82, *SD* = 1.29) and did not differ from the other two conditions.

#### Hypothesis Testing via Indirect Effects

Two parallel-process models testing indirect effects were constructed using PROCESS v4.2 (Hayes, 2022). Support for multicultural policy and ideology were outcome variables. Like Study 1, national identification was the predictor variable and individual responsibility belief was the intermediary variable in both models. Ethnic conception of national identity was an additional intermediary variable to test if the indirect effect through individual responsibility belief remained significant when the same effect of ethnic conception of national identity was accounted for. While the conditions were originally included as moderators of the relationship between national identification and individual responsibility belief, the contrast-coded interaction effects were non-significant, suggesting there were no differences between conditions in our hypothesized model (despite that the interactions were slightly in the expected direction). We therefore report the model below independently of experimental conditions, and examine citizenship as a moderator of both H1a and H1b to check if the model applies specifically for national majority members, as in Study 2. Covariates included age and gender.

Our hypothesis was confirmed for Belgian nationals only. A significant interaction was found between citizenship and national identification when predicting the belief in individual responsibility, *B* = .17, *SE* = .07, *95% CI* [0.04, 0.31], *t*(353) = 2.51, *p* = .013, Δ*R^2^* = 0.02. Consistent with H1a, simple effects showed that the positive relationship between national identification and individual responsibility belief was significant only among Belgian nationals, *B* = .17, *SE* = .05, *95% CI* [0.08, 0.26], *t*(353) = 3.55, *p* < .001, but not among participants with other nationalities, *B* = –.00, *SE* = .05, *95% CI* [–0.10, 0.09], *t*(353) = –0.09, *p* = .926. Therefore, highly identified national majority members were most likely to express a strong belief in individual responsibility. By contrast, national identification was not associated with an ethnic conception of national identity, neither as a main effect, nor as an interaction with citizenship, *B* = .13, *SE* = .09, *95% CI* [–0.05, 0.31], *t*(353) = 1.46, *p* = .146.

Consistent with H1b, a stronger belief in individual responsibility was related to significantly lower support for multicultural policy as a main effect, *B* = –.22, *SE* = .10, 95% CI [–0.43, –0.02], *t*(350) = –2.17, *p* = .031; *d* = 0.23. The interaction between citizenship and individual responsibility belief was non-significant, *B* = –.15, *SE* = .14, 95% CI [–0.42, 0.12], *t*(350) = –1.09, *p* = .278, although the direction suggested the effect was more pronounced among Belgian nationals. No such main or interaction effects were significant when predicting support for multicultural ideology, *B* = –.02, *SE* = .08, 95% CI [–0.18, 0.13], *t*(350) = –0.31, *p* = .757, *B* = –.05, *SE* = .11, 95% CI [–0.26, 0.15], *t*(350) = –0.51, *p* = .611, respectively. Therefore, the more participants believed individuals were responsible for their own fate, the less they supported a concrete (redistributive) facet of multiculturalism. This occurred when controlling for ethnic conception of national identity that was neither associated with support for multicultural policy, *B* = .03, *SE* = .08, 95% CI [–0.12, 0.18], *t*(350) = 0.37, *p* = .711, nor ideology, 95% CI [–0.09, 0.08], *t*(280) = –0.08, *p* = .937.

When considering the indirect effect of national identification on support for multicultural policy through individual responsibility belief, the index of moderated mediation, by citizenship, was significant, *B* = –.06, *SE* = .03, 95% CI [–0.12, –0.01]. Indeed, the indirect effect of national identification on support for multicultural policy through the belief in individual responsibility was significant specifically among those with Belgian citizenship only, *B* = –.06, *SE* = .03, 95% CI [–0.12, –0.02], and not among those with other nationalities, *B* = .00, *SE* = .01, 95% CI [–0.02, 0.03]. No indirect effects were significant when predicting support for multicultural ideology. Therefore, the more national majorities felt connected to Belgium, the greater their individual responsibility belief, and the more they were opposed to multicultural policy, but not ideology.

### Discussion

Study 4 revealed that the hypothesized conceptual model is also at least partially relevant in Belgium, specifically among the national majority group, and specifically when support for multicultural policy (but not ideology) was the outcome variable. Similar to Study 2, the lack of indirect effect on support for multicultural ideology may be explained by the fact that political endorsement of cultural diversity is generally more pronounced in Belgium than in Switzerland (Banting & Kymlicka, 2013). This institutional support for diversity, coupled with Belgium’s history of colonialism, may explain why individual justice beliefs were not associated with opposition to multicultural ideology, but with policy. Moreover, the indirect effect on support for multicultural policy occurred above and beyond an ethnic conception of Belgian national identity which was not significantly associated, perhaps because Belgian national identity is not ethnically defined, or at least this specific aspect of national identity does not organize peoples’ attitudes towards multiculturalism. Importantly, this result suggests it is indeed a belief in individual justice that appears to drive opposition to multicultural policy by individuals who strongly identify with their country, rather than an ‘us versus them’ intergroup logic.

## General Discussion

Using correlational, experimental and survey approaches, the four studies in this paper support the main hypothesis that national identification with Western liberal countries is associated with stronger beliefs in individual justice, which in turn fuel opposition to multiculturalism—especially multicultural policy. We thereby offer a perspective on multicultural attitudes that emphasizes the role of justice beliefs derived from identification with (majority) national groups.

In all four studies, a belief in individual responsibility (or in the benefits of hard work) was associated with a rejection of multiculturalism. In Switzerland, this was the case regardless of the abstract or concrete facet of multiculturalism under scrutiny. In contexts in which cultural diversity was normatively valued—arguably in the experimental paradigm in Study 2 and in Belgium in Study 4—the belief in individual responsibility was associated with a rejection of concrete multicultural policies, but not of its abstract principles.

Our reasoning is based on the general conjecture that in classically liberal societies, the ethos of individual responsibility is anchored, on the individual level, in identification with the national community. Research has shown that in addition to shaping a sense of perceived threat (Badea et al., 2018; Green & Staerklé, 2023; Verkuyten, 2009), the *content* of national identity is a critical factor for understanding attitudes towards newcomers and policies addressing cultural diversity (Pehrson & Green, 2010; Reijerse et al., 2015; Reynolds et al., 2015; Yogeeswaran & Dasgupta, 2014a). This past research has focused on citizenship regimes and the degree to which foreigners are included or excluded in the national community. In this paper, we argue that (justice) beliefs associated with national identities should be given greater attention in studies on attitudes towards multiculturalism.

Results from Studies 1, 3, and 4 showed that national identification was associated with a stronger belief in individual responsibility. This is consistent with the idea that national identification in Western societies is associated with the classically liberal, atomistic belief that ‘we are all individuals’ (Jetten et al, 2002) and with the tendency in Western liberal countries to attribute individuals’ behavior and choices to their internal characteristics rather than to situational factors (Ross, 1977). Switzerland was an ideal context to test our hypotheses as it is a prototypically classically liberal country, much like the United Kingdom or the United States, where norms of individual responsibility are pervasive. However, comparative research has also shown that Switzerland offers only weak institutional support for cultural diversity (Banting & Kymlicka, 2013). This is unlike many (economically) liberal *settler* societies, and contrasts with other European countries such as Belgium, where institutional support for cultural diversity is more extensive. The fact that the conceptual model was replicated in Belgium, at least for predicting support for multicultural policy, increases our confidence in the reliability and replicability of the effects. The fact that the model was not replicated when predicting support for multicultural ideology in Belgium illustrates that variation may exist in contexts in which a normative emphasis on individuals is coupled with greater normative endorsement of diversity; and in which individual justice beliefs are less associated with opposition to multicultural ideology. Nevertheless, future research should continue to investigate boundary conditions of the negative relationship between the belief in individual responsibility and support for multiculturalism (either as an abstract ideology or as a concrete policy) among national majority members. According to our theorizing, this relationship may vary depending on the degree to which values and norms of individual freedom and responsibility and cultural diversity are embedded in national self-concepts, thereby shaping the way majority members view multiculturalism and collective conceptions of justice (see also Gale et al., 2021).

The empirical focus of Studies 1 through 3 on Switzerland (including a fictitious society paradigm in Study 2) raises the question as to whether the effect of individual responsibility belief was confounded with restrictive immigration attitudes and/or prejudice towards cultural minority groups. In Study 1, we did find that the belief in individual responsibility played a role that was above and beyond other status legitimizing ideologies. Furthermore, in Study 3, we found that the belief in hard work played a role above and beyond restrictive immigration attitudes and perceived entitlement of nationals over newcomers. In the Belgian context, we found the effect was present above and beyond an ethnic conception of national identity. We therefore argue that this belief implies that immigrants and other cultural minorities are not only perceived as (threatening or dangerous) group members, but also (and maybe foremostly) as *individuals* who are *undeserving* of recognition and redistribution; a view that appears to be relatively ‘universal’ across Europe (Laenen & Meuleman, 2017; van Oorschot, 2006) and that has also been put forward in research on symbolic racism (see Sears & Henry, 2003). As a result, national majorities with high levels of national identification oppose multiculturalism not only with intergroup prejudice always in mind, but also because they view multiculturalism (emphasizing groups) as incompatible with their fundamental, taken-for-granted, (individual) conception of justice. This distinction is crucial for understanding the origins of opposition to multiculturalism, as occasional misconceptions of intentions (e.g., across ideological lines or sources of threat; Rios, 2022) may hinder productive communication to face the challenges of cultural diversity.

These studies had limitations. First, convenience samples were used in Studies 1, 2, and 4, and the sample sizes were relatively small in Studies 1 and 2 (albeit within the needed range). Study 3 offset this limitation with a strongly powered, nationally representative sample in Switzerland. Given that secondary survey data was used, measures in this study were comparable, but different from those in studies 1, 2, and 4. Second, the expected causality in our model was only partially supported through our experimental studies. Study 2 provided support for causality between the shared belief in individual responsibility and opposition to multiculturalism. This occurred when predicting multicultural policy, with a plausible explanation as to why there was no effect on multicultural ideology. However, we did not manipulate the independent variable of national identification. When we manipulated the extent to which the belief in individual responsibility *defined* national identification (Study 4), results were in the expected direction but non-significant. Importantly, existing research does suggest national identification is a lens through which multiculturalism is perceived (Verkuyten, 2009). National identification should thus shape justice conceptions rather than the other way around. It may be that endorsing a shared belief in individual responsibility could lead an individual to identify more strongly with their country, although this would only plausibly be the case if that country were already characterized by normative beliefs in individual responsibility, bringing the primacy of national identification back to the forefront. Indeed, notwithstanding these limitations, the remarkably coherent pattern of findings across the four studies, specifically among national majorities, provides evidence for our reasoning.

## Conclusion

The present research offers a social justice perspective of multicultural attitudes among national majorities. Extensive prior research has shown that national majority attitudes towards multiculturalism and immigration can be explained by a sense of threat to their national identity (Badea et al., 2018; Green & Staerklé, 2023; Verkuyten, 2009), especially in exclusionary national contexts (Pehrson & Green, 2010; Yogeeswaran & Dasgupta, 2014a). Evidence of the role of perceived threat is indeed well established in the literature (see also Iyer, 2022). Yet some of these studies are potentially marred by a relative proximity between predictor (perceived threat from immigrants) and outcome variables (attitudes towards immigration and multicultural policies). Our approach avoids this potential tautological pitfall, since attitudes towards multiculturalism are explained by justice conceptions that are conceptually and semantically independent from the target population (the justice items never mention immigrants or cultural minority groups).

Other research has demonstrated that opposition to policies that redress group-based inequalities (such as affirmative action policies) can be explained by the perception that these policies violate principles that people hold dear, such as the individual justice principles of merit and personal responsibility (Bobocel et al., 1998; Son Hing et al., 2011). These findings are in line with our own justice-based approach as we concur that justice-based concerns are a key factor in understanding attitudes towards multicultural ideology and policies. We contribute to this literature by showing that these justice conceptions should not be seen as simple individual differences but rather should be integrated in a normative framework accounting for the ideological origins of these justice conceptions, namely identification with classically liberal countries. By bridging national identity and social justice lines of research, our approach thus suggests that for national majorities, perceived incompatibility between multiculturalism (collective justice) and individual responsibility (individual justice) is based on the endorsement of culturally determined and ideologically dominant national norms of individual responsibility. From a practical perspective, exploring how to reconcile norms of individual and collective justice (e.g., through media portayals, adjusting diversity policies, dialogue and dialectical thinking, or other means) could provide actionable recommendations for fostering intergroup harmony and addressing opposition to multiculturalism in liberal societies. We hope that this justice-based perspective on multiculturalism may open fruitful avenues for future research that seek to gain a better understanding of why the integration of minority groups in host societies often encounters fierce resistance.

## Data Accessibility Statement

The data and material associated with the studies in this article are available at https://osf.io/3j46b/?view_only=7fc894f8eb1346e1b3ce368ff4a0f19d.

## Additional File

The additional file for this article can be found as follows:

10.5334/irsp.941.s1 Supplementary File 1. National identification manuscript.

## Footnotes

‘Liberalism’ here refers to classical liberal enlightenment philosophy that emphasizes individual autonomy and responsibility (see Hayek, 2005), and should not be confounded with everyday usage of the term in particular in the context of U.S. politics (where it is opposed to ‘conservatism’).

The same data file was used in Gale and Staerklé (2019; Study 1). In the present study, a subsample and additional measures were used. For a list of all measures in the data file, see Gale & Staerklé (2019).

Power analyses were conducted using G*Power 3.1, based on previously documented associations between national identification and rejection of multiculturalism (Verkuyten, 2009). The effect sizes (r) in these studies were .21 (Study 1), .37 (Study 2), and .36 (Study 3). To replicate these effects with .80 power, a sample between 52 and 173 participants was needed.

Analyses reported in all studies include covariates. When covariates were excluded, conclusions were the same.

When Social Dominance Orientation (SDO), right-wing authoritarianism, and political orientation were included in the models as additional intermediary variables, the negative indirect effect of individual responsibility belief remained significant in addition to SDO, in both models.

Based on effects from Study 1, a power analysis using G*Power 3.1 showed that 79 participants were needed to replicate the effect of individual responsibility on support for multicultural policy, and 122 were needed for the same effect on support for multicultural ideology. We therefore had enough participants in terms of full sample size and just about enough ‘native’ participants to replicate the effect on support for multicultural policy.

Two more items were included that expressed a more prescriptive belief in individual responsibility (‘Individuals should be responsible for their own outcomes,’ ‘People should be themselves responsible to progress’; *r* = .77). When controlling for age and gender, a one-way analysis of covariance showed that participants in the high individual responsibility condition (*M* = 4.73, *SE* = 0.13) scored marginally higher on this measure than those in the low individual responsibility condition (*M* = 4.40, *SE* = 0.13), *F*(1, 165) = 3.17, *p* = .077, η_p_^2^ = 0.02. Furthermore, this variable was positively associated with the existing measure of belief in individual responsibility (*r* = .57) and negatively with support for multicultural policy (*r* = –.31), with no relationship with support for multicultural ideology (*r* = –.07).

Nevertheless, 16.5% (*n* = 205) of the sample did indicate they had at least one parent who had immigrated to Switzerland. Conclusions were the same when these participants were excluded (*N* = 1,096 after exclusions).

We also tested this model controlling for restrictive immigration policy support and a preference for national in-group members on the job market. Conclusions remained unchanged. Moreover, because the PROCESS macro (Hayes, 2022) cannot take weights into account, reported analyses were replicated with weighted data using MPLUS version 5.1 (Muthén & Muthén, 1998–2012). Results remained consistent.

We aimed to recruit approximately 100 participants per experimental condition (see preregistration https://aspredicted.org/W9N_C6S). A sensitivity power analysis conducted using G*Power suggested that with this sample size an effect of *R^2^* = .02 could be detected (.80 power, α = .05).

Inter-item reliability for this measure (not sensitive to the number of items) was *r* = .21, which remains low but within the acceptable range of .20–.40 (Briggs & Cheek, 1986).

## References

[1] Ariely, G. (2012). Do those who identify with their nation always dislike immigrants?: An examination of citizenship policy effects. Nationalism and Ethnic Politics, 18, 242–261. 10.1080/13537113.2012.680862

[2] Azzi, A. E. (1992). Procedural justice and the allocation of power in intergroup relations: Studies in the United States and South Africa. Personality and Social Psychology Bulletin, 18, 736–747. 10.1177/0146167292186010

[3] Badea, C., Iyer, A., & Aebischer, V. (2018). National identification, endorsement of acculturation ideologies and prejudice: The impact of the perceived threat of immigration. International Review of Social Psychology, 31, 1–10. 10.5334/irsp.147

[4] Banting, K., & Kymlicka, W. (2013). Is there really a retreat from multiculturalism policies? New evidence from the multiculturalism policy index. Comparative European Politics, 11, 577–598. 10.1057/cep.2013.12

[5] Berry, J. W., & Kalin, R. (1995). Multicultural and ethnic attitudes in Canada: An overview of the 1991 national survey. Canadian Journal of Behavioural Science, 27, 301–320. 10.1037/0008-400X.27.3.301

[6] Bobocel, D. R., Son Hing, L. S., Davey, L. M., Stanley, D. J., & Zanna, M. P. (1998). Justice-based opposition to social policies: Is it genuine? Journal of Personality and Social Psychology, 75, 653–669. 10.1037/0022-3514.75.3.653

[7] Briggs, S. R., & Cheek, J. M. (1986). The role of factor analysis in the development and evaluation of personality scales. Journal of Personality, 54, 106–148. 10.1111/j.1467-6494.1986.tb00391.x

[8] Brubaker, W. R. (1990). Immigration, citizenship and the nation-state in France and Germany: A comparative historical analysis. International Sociology, 5, 379–407. 10.1177/026858090005004003

[9] Deschamps, J.-C. (1982). Social identity and relations of power between groups. In H. Tajfel (Ed.), Social identity and intergroup relations (pp. 85–98). Cambridge University Press.

[10] Devos, T., & Mohamed, H. (2014). Shades of American identity: Implicit relations between ethnic and national identities. Social and Personality Psychology Compass, 8, 739–754. 10.1111/spc3.12149 27011765 PMC4800470

[11] Esses, V. M., Wagner, U., Wolf, C., Preiser, M., & Wilbur, C. J. (2006). Perceptions of national identity and attitudes toward immigrants and immigration in Canada and Germany. International Journal of Intercultural Relations, 30(6), 653–669. 10.1016/j.ijintrel.2006.07.002

[12] Falomir-Pichastor, J. M., & Frederic, N. S. (2013). The dark side of heterogeneous ingroup identities: National identification, perceived threat, and prejudice against immigrants. Journal of Experimental Social Psychology, 49, 72–79. 10.1016/j.jesp.2012.08.016

[13] Gale, J., & Staerklé, C. (2019). Multiculturalism in classically liberal societies: Group membership and compatibility between individual and collective justice. Journal of Experimental Social Psychology, 85. 10.1016/j.jesp.2019.103877

[14] Gale, J., Staerklé, C., Green, E. G. T., & Visintin, E. P. (2021). Multicultural attitudes in Europe: A multilevel analysis of perceived compatibility between individual and collective justice. Journal of Social and Political Psychology, 9, 419–437. 10.5964/jspp.7081

[15] Gale, J., & Yogeeswaran, K. (2024). Perceiving multiple truths: Does dialectical thinking harmonize colourblind and multicultural ideals? British Journal of Psychology, 115, 454–471. 10.1111/bjop.12697 38240692

[16] Gale, J., & Yogeeswaran, K. (2025). A colorblind ideal and the motivation to improve intergroup relations: The role of an (in)congruent status quo. Journal of Experimental Social Psychology, 116. 10.1016/j.jesp.2024.104693

[17] Green, E. G. T., & Staerklé, C. (2023). Migration and multiculturalism. In L. Huddy, D. O. Sears, J. S. Levy, & J. Gerit (Eds.), Oxford handbook of political psychology (3rd ed.). Oxford University Press. 10.1093/oxfordhb/9780197541302.013.27

[18] Guimond, S., de la Sablonnière, R., & Nugier, A. (2014). Living in a multicultural world: Intergroup ideologies and the societal context of intergroup relations. European Review of Social Psychology, 25, 142–188. 10.1080/10463283.2014.957578

[19] Hayek, F. A. (2005). The road to serfdom with the intellectuals and socialism. The condensed version of the road to serfdom as it appeared in the April 1945 edition of Reader’s Digest. Institute of Economic Affairs.

[20] Hayes, A. F. (2022). Introduction to mediation, moderation and conditional process analysis: A regression-based approach (3rd ed.). The Guilford Press.

[21] Iacoviello, V., & Lorenzi-Cioldi, F. (2015). Individualistic tendencies: When group status makes the difference. Group Processes and Intergroup Relations, 18, 540–556. 10.1177/1368430214552332

[22] Iyer, A. (2022). Understanding advantaged groups’ opposition to diversity, equity, and inclusion (DEI) policies: The role of perceived threat. Social and Personality Psychology Compass, 16, e12666. 10.1111/spc3.12666

[23] Jetten, J., Postmes, T., & McAuliffe, B. (2002). “We are all individuals”: Group norms of individualism and collectivism, levels of identificatication, and identity threat. Journal of Social Psychology, 32, 189–207. 10.1002/ejsp.65

[24] Joffe, H., & Staerkle, C. (2007). The centrality of the self-control ethos in western aspersions regarding outgroups: A social representational approach to stereotype content. Culture and Psychology, 13, 395–418. 10.1177/1354067X07082750

[25] Joppke, C. (2004). The retreat of multiculturalism in the liberal state: Theory and policy. The British Journal of Sociology, 55, 237–257. 10.1111/j.1468-4446.2004.00017.x 15233632

[26] Kukathas, C. (2003). The liberal archipelago: A theory of diversity and freedom. Oxford University Press. 10.1093/019925754X.001.0001

[27] Kymlicka, W. (2013). Neoliberal multiculturalism? In P. A. Hall & M. Lamont (Eds.), Social Resilience in the Neoliberal Era (pp. 99–125). Cambridge University Press. 10.1017/CBO9781139542425.007

[28] Laenen, T., & Meuleman, B. (2017). A universal rank order of deservingness? Geographical, temporal and social-structural comparisons. In The social legitimacy of targeted welfare: Attitudes on welfare deservingness (pp. 37–54). Edward Elgar. 10.4337/9781785367212.00012

[29] Major, B., Gramzow, R. H., McCoy, S. K., Levin, S., Schmader, T., & Sidanius, J. (2002). Perceiving personal discrimination: The role of group status and legitimizing ideology. Journal of Personality and Social Psychology, 82, 269–282. 10.1037//0022-3514.82.3.269 11902616

[30] Martinovic, B., & Verkuyten, M. (2013). “We were here first, so we determine the rules of the game”: Autochthony and prejudice towards out-groups. European Journal of Social Psychology, 43, 637–647. 10.1002/ejsp.1980

[31] Meeus, J., Duriez, B., Vanbeselaere, N., & Boen, F. (2010). The role of national identity representation in the relation between in-group identification and out-group derogation: Ethnic versus civic representation. British Journal of Social Psychology, 49, 305–320. 10.1348/014466609X451455 19558752

[32] Moghaddam, F. M. (2008). Multiculturalism and intergroup relations: Psychological implications for democracy in global context. American Psychological Association. 10.1037/11682-000

[33] Müller, J.-W. (2016). What is populism? University of Pennsylvania Press. 10.9783/9780812293784

[34] Muthén, L. K., & Muthén, B. O. (1998–2012). Mplus User’s Guide (Seventh Ed). Muthén & Muthén.

[35] Pehrson, S., & Green, E. G. T. (2010). Who we are and who can join us: National identity content and entry criteria for new immigrants. Journal of Social Issues, 66(4), 695–716. 10.1111/j.1540-4560.2010.01671.x

[36] Plaut, V. C., Garnett, F. G., Buffardi, L. E., & Sanchez-Burks, J. (2011). “What about me?” Perceptions of exclusion and whites’ reactions to multiculturalism. Journal of Personality and Social Psychology, 101, 337–53. 10.1037/a0022832 21534702

[37] Politi, E., Chipeaux, M., Lorenzi-Cioldi, F., & Staerklé, C. (2020). More royalist than the king? Immigration policy attitudes among naturalized citizens. Political Psychology, 41, 607–625. 10.1111/pops.12642

[38] Postmes, T., Haslam, S. A., & Jans, L. (2013). A single-item measure of social identification: Reliability, validity, and utility. British Journal of Social Psychology, 52, 597–617. 10.1111/bjso.12006 23121468

[39] Reicher, S. D., Spears, R., & Haslam, S. A. (2010). The social identity approach in social psychology. In M. S. Wetherell & C. T. Mohanty (Eds.), The SAGE handbook of identities (pp. 45–62). SAGE. 10.4135/9781446200889.n4

[40] Reijerse, A., Vanbeselaere, N., Duriez, B., & Fichera, G. (2015). Accepting immigrants as fellow citizens: Citizenship representations in relation to migration policy preferences. Ethnic and Racial Studies, 38, 700–717. 10.1080/01419870.2014.916812

[41] Reynolds, K. J., Batalha, L., & Subasic, E. (2015). The social psychology of social (dis)harmony: Implications for political leaders and public policy. In J. P. Forgas, K. Fiedler, & B. Crano (Eds.), Social psychology and politics (pp. 337–356). Psychology Press. 10.4324/9780203846957

[42] Riaño, Y., & Wastl-Walter, D. (2006). Immigration policies, state discourses on foreigners, and the politics of identity in Switzerland. Environment and Planning A, 38, 1693–1713. 10.1068/a37411

[43] Rios, K. (2022). Multiculturalism and colorblindness as threats to the self: A framework for understanding dominant and non-dominant group members’ responses to interethnic ideologies. Personality and Social Psychology Review, 26, 315–341. 10.1177/10888683221093130 35620828

[44] Ross, L. (1977). The intuitive psychologist and his shortcomings: Distortions in the attribution process. In L. Berkowitz (Ed.), Advances in experimental social psychology (pp. 173–220). Academic Press. 10.1016/S0065-2601(08)60357-3

[45] Ryan, C. S., Hunt, J. S., Weible, J. A., Peterson, C. R., & Casas, J. F. (2007). Multicultural and colorblind ideology, stereotypes, and ethnocentrism among black and white americans. Group Processes & Intergroup Relations, 10, 617–637. 10.1177/1368430207084105

[46] Schwiter, K. (2013). Neoliberal subjectivity: Difference, free choice and individualised responsibility in the life plans of young adults in Switzerland. Geographica Helvetica, 68, 153–159. 10.5194/gh-68-153-2013

[47] Sears, D. O., Citrin, J., Cheleden, S. V., & van Laar, C. (1999). Cultural diversity and multiculturalism: Is ethnic balkanization psychologically inevitable? In D. A. Prentice & D. T. Miller (Eds.), Cultural divides: Understanding and overcoming group conflict (pp. 35–79). Russell Sage Foundation.

[48] Sears, D. O., & Henry, P. J. (2003). The origins of symbolic racism. Journal of Personality and Social Psychology, 85, 259–275. 10.1037/0022-3514.85.2.259 12916569

[49] Shweder, R. A., Much, N. C., Mahapatra, M., & Park, L. (1997). The “big three” of morality (autonomy, community, divinity) and the “big three” explanations of suffering. In A. Brandt & P. Rozin (Eds.), Morality and Health (pp. 119–169). Routledge.

[50] Smeekes, A., Verkuyten, M., & Martinovic, B. (2015). Longing for the country’s good old days: National nostalgia, autochthony beliefs, and opposition to Muslim expressive rights. British Journal of Social Psychology, 54, 561–580. 10.1111/bjso.12097 25430971

[51] Son Hing, L. S., Bobocel, D. R., Zanna, M. P., Garcia, D. M., Gee, S. S., & Orazietti, K. (2011). The merit of meritocracy. Journal of Personality and Social Psychology, 101, 433–450. 10.1037/a0024618 21787093

[52] Soutphommasane, T. (2005). Grounding multicultural citizenship: From minority rights to civic pluralism. Journal of Intercultural Studies, 26, 401–416. 10.1080/07256860500270239

[53] Staerklé, C. (2009). Policy attitudes, ideological values and social representations. Social and Personality Psychology Compass, 3, 1096–1112. 10.1111/j.1751-9004.2009.00237.x

[54] Staerklé, C., & Green, E. G. T. (2018). Right-wing populism as a social representation: A comparison across four European countries. Journal of Community & Applied Social Psychology, 28, 430–445. 10.1002/casp.2369

[55] Tajfel, H., & Turner, J. (1979). An integrative theory of intergroup conflict. In W. G. Austin & S. Worchel (Eds.), The social psychology of intergroup relations (pp. 33–47). Brooks/Cole.

[56] The Heritage Foundation. (2023). Index of economic freedom. Retrieved from www.heritage.org/index

[57] Tyler, T. R., & van der Toorn, J. (2013). Social justice. In L. Huddy, D. O. Sears, & J. S. Levy (Eds.), Oxford handbook of political psychology (2nd ed., pp. 627–661). Oxford University Press. 10.1093/oxfordhb/9780199760107.013.0020

[58] van Oorschot, W. (2006). Making the difference in social Europe: Deservingness perceptions among citizens of European welfare states. Journal of European Social Policy, 16(1), 23–42. 10.1177/0958928706059829

[59] Verkuyten, M. (2009). Support for multiculturalism and minority rights: The role of national identification and out-group threat. Social Justice Research, 22, 31–52. 10.1007/s11211-008-0087-7

[60] Wimmer, A. (2011). A Swiss anomaly? A relational account of national boundary-making. Nations and Nationalism, 17, 718–737. 10.1111/j.1469-8129.2011.00517.x

[61] Yogeeswaran, K., & Dasgupta, N. (2014a). Conceptions of national identity in a globalised world: Antecedents and consequences. European Review of Social Psychology, 25, 189–227. 10.1080/10463283.2014.972081

[62] Yogeeswaran, K., & Dasgupta, N. (2014b). The devil is in the details: Abstract versus concrete construals of multiculturalism differentially impact intergroup relations. Journal of Personality and Social Psychology, 106, 772–89. 10.1037/a0035830 24611896

