# Supplementary material for: Identifying with a Classically Liberal Nation: A Social Justice Perspective on Majority Opposition to Multiculturalism
Source: Int Rev Soc Psychol. 2025 Mar 19;38:2. doi: 10.5334/irsp.941 (PMC12372792; doi:10.5334/irsp.941)
Supplement: Supplementary File 1. — National identification manuscript. [file irsp-38-941-s1.pdf]

Identifying with a Classically Liberal Nation: A Social Justice Perspective on Majority Opposition to Multiculturalism

Supplementary File 1

Study 1 Measures

National identification (4 items):

1. "I feel Swiss,"
2. "I feel committed to Switzerland,"
3. "I identify with Switzerland,"
4. "I feel united with the Swiss."

Individual responsibility belief (4 items):

1. "Switzerland is a society where each individual can attain a better position in society,"
2. "Some people cannot manage to progress in society because of social inequalities" (reverse-coded),
3. "Most people who do not progress should not blame the system; they are responsible themselves,"
4. "Some people struggle to attain a better position in society because of their origins" (reverse-coded).

Support for multicultural ideology (4 items):

1. "Cultural affiliation is a precious distinction between individuals that should be valued,"
2. "It is important to remember that we are shaped by the cultural groups to which we belong,"
3. "Concentrating on cultural and ethnic differences allows us to learn more about others and to mutually appreciate each other,"
4. "In general, cultural differences should be celebrated."

Support for multicultural policy (3 items):

1. "Schools with a large proportion of children from cultural minority groups should receive special funding in order to guarantee conditions which favor success,"
2. "In order to guarantee a certain diversity between employees, positions should be reserved for qualified members of cultural minority groups,"
3. "The government should set an example and hire more cultural minority group members."

A Principal Component Analysis showed that items of the ideology and policy measures of support for multiculturalism loaded on distinct dimensions (Eigenvalues: $\lambda_{ideology} = 3.37$ and $\lambda_{policy} = 1.21$, total variance explained: 65%).

Study 2 Measures

National identification (1 item):

1. "I identify with the island of Ral,"

Individual responsibility belief (2 items):

1. “Switzerland is a society where each individual can attain a better position in society,”
2. “Most people who do not progress should not blame the system; they are responsible themselves,”

Support for multicultural ideology (5 items):
1. “Cultural affiliation is a precious distinction between individuals that should be valued,”
2. “It is important to remember that we are shaped by the cultural groups to which we belong,”
3. “Concentrating on cultural and ethnic differences allows us to learn more about others and to mutually appreciate each other,”
4. “In general, cultural differences should be celebrated.”
5. “The cohesion of this island is reinforced by recognizing and valuing different cultural groups.”

Support for multicultural policy (4 items):
1. “Schools with a large proportion of children from cultural minority groups should receive special funding in order to guarantee conditions which favor success,”
2. “In order to guarantee a certain diversity between employees, positions should be reserved for qualified members of cultural minority groups,”
3. “The government should set an example and hire more cultural minority group members.”
4. “Universities should make special efforts to recruit and support students from cultural minority groups.”

A Principal Component Analysis showed that items of the two measures of support for multiculturalism loaded on distinct dimensions (Eigenvalues: λ_{policy} = 3.00 and $\lambda_{ideology}$ = 1.57, total variance explained: 51%).

Study 2 Supplementary Analyses

To test our hypotheses when including citizenship as a moderator, we calculated two supplementary models that were identical to those reported in the manuscript, except that the three citizenship groups were separated in these models using contrast codes. Indeed, when non-Swiss participants were separated from naturalized and dual- (or triple-) citizens, the interaction between experimental conditions and the contrast codes (distinguishing first Swiss only participants (coded .67) from the others (coded .33), and distinguishing second non-Swiss participants (coded -.5) from naturalized and dual-citizens (coded .5)) revealed again a significant interaction between Swiss only and everyone else, *B* = -.35, *SE* = .18, *95% CI* [-0.70, -0.00], *t*(161) = -1.97, *p* = .021, ΔR^2 = 0.03, and a non-significant interaction between non-Swiss participants and naturalized and dual citizens, *B* = .15, *SE* = .24, *95% CI* [-0.33, 0.62], *t*(161) = 0.60, *p* = .547, suggesting participants with Swiss citizenship only were indeed the ones for whom support for multicultural policy differed by experimental condition.

Study 4 Supplementary Analyses

Like Study 2, two additional models were calculated that were identical to those reported in the manuscript, except that the three citizenship groups were separated in these

additional models using contrast codes. In this way, Belgian-only participants (coded .67) were distinguished from the others (coded -.33), and non-Belgian participants (coded -.5) were distinguished from dual-citizens (coded .5). When predicting support for multicultural policy, a significant index of moderated mediation was revealed for the contrast code comparing Belgian citizenship only to everyone else, B = -.06, SE = .03, 95% CI [-0.12, -0.01], but not for the contrast code comparing non-Belgians to dual/triple citizens, B = -.06, SE = .05, 95% CI [-0.17, 0.03]. The indirect effect of national identification on support for multicultural policy through the belief in individual responsibility was significant among those with Belgian citizenship only, B = -.06, SE = .03, 95% CI [-0.12, -0.02] and not among dual citizens, B = -.00, SE = .01, 95% CI [-0.04, 0.03], nor non-Belgians, B = .00, SE = .05, 95% CI [-0.09, 0.10]. This suggests the model was specifically pertinent among participants with Belgian citizenship only.
